# Ciprofol: A Novel Alternative to Propofol in Clinical Intravenous Anesthesia?

**DOI:** 10.1155/2023/7443226

**Published:** 2023-01-19

**Authors:** Ming Lu, Jian Liu, Xikun Wu, Zhiqing Zhang

**Affiliations:** Department of Pharmacy, The Second Hospital of Hebei Medical University, Shijiazhuang, Hebei Province 050000, China

## Abstract

Ciprofol is a novel compound that was independently developed in China. According to the Chinese product instructions approved by the China National Medical Products Administration and the information of official website, indications for ciprofol include sedation and anesthesia during the surgical/procedure of nontracheal intubation, induction and maintenance of general anesthesia, and sedation during intensive care. Ciprofol is a short-acting intravenous sedative based on the structural modification of propofol. Ciprofol has high efficacy, good selectivity, and fewer adverse reactions, indicating good clinical application potential. A series of clinical studies have been conducted to evaluate the sedative effect of ciprofol in various procedures and settings, including gastroscopy and colonoscopy, fiber-optic bronchoscopy, general anesthesia in elective surgeries, and mechanical ventilation in intensive care units. This review summarizes the chemical structure, pharmacodynamics, and pharmacokinetic properties of ciprofol. We also assessed the efficacy and safety of ciprofol by synthesizing the relevant clinical trial data.

## 1. Introduction

Anesthesia is an effective option to relieve tension and pain in patients undergoing procedures to ensure smooth progress of the examination and operation [1]. Among the various forms of anesthesia, general anesthesia via intravenous drug administration has been widely used because of its rapid action, low irritation, and ease of control [2, 3]. Propofol is the most commonly used short-acting intravenous anesthetic. Propofol is an allosteric potentiator and agonist of the gamma-aminobutyric acid type A (GABA_A_) receptor [4]. Propofol exerts sedative and general anesthetic effects by reducing excitability and enhancing the central inhibitory GABA neurotransmitter [5, 6]. Propofol has been widely recognized in clinical practice, benefiting from its pharmacodynamic and pharmacokinetic (PK) profiles, rapid onset of anesthesia, rapid recovery, almost complete lack of residual effects, and absence of respiratory irritation [7, 8]. However, various adverse drug reactions (ADRs) related to propofol can occur, including local pain during induction, cardiovascular and respiratory depression, potential headache, nausea and vomiting during resuscitation, and a drop in blood pressure due to rapid injection [9–12]. Furthermore, propofol infusion syndrome (PRIS) is a rare but potentially lethal ADR to propofol. PRIS can cause metabolic disorders, organ system failure, and death [13–15]. Given these limitations, attempts have been made to transform and modify its core structure to design new compounds with lower ADR incidence.

Ciprofol (HSK3486) was developed by Haisco Pharmaceutical Group Co., Ltd. (Chengdu, China) and was first reported in 2017 [16, 17]. The chemical structure of ciprofol is (R)-2-(1-cyclopropyl ethyl)-6-isopropylphenol. Ciprofol was approved by the China National Medical Products Administration (NMPA) (http://www.nmpa.gov.cn) for sedation during gastrointestinal endoscopy on December 15, 2020, after a priority review. Subsequently, ciprofol was approved for sedation during bronchoscopy, induction, and maintenance of general anesthesia. A new indication for ciprofol was recently approved by the NMPA for sedation during intensive care. According to the information of *Clinicaltrials.gov*, a phase III clinical trial evaluating the efficacy and safety of ciprofol injection for the induction of sedation/anesthesia in subjects undergoing gynecological outpatient surgeries has been completed in China, and this indication is in review and approval stage at present. Ciprofol is expected to continue to expand the scope of clinical applications. This manuscript describes the chemical structure, pharmacodynamics, and PK properties of ciprofol and summarizes its efficacy and safety evaluations.

## 2. Structural Features and Mechanisms

**Figure 1:**
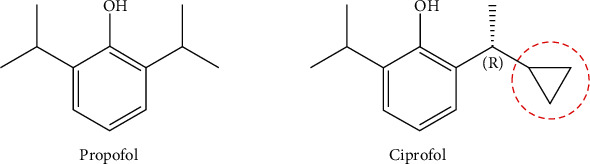
Chemical structures of propofol and ciprofol.

GABA_A_ receptors exhibit stereoselectivity in anesthetics [18]. The core structure of classic short-acting intravenous anesthetics is 2, 6-disubstituted phenol, which binds to the GABA_A_ receptor to produce an anesthetic effect. Propofol is the most widely used of these drugs. Ciprofol adds a cyclopropyl group to the side chain of the core structure (Figure 1). The addition of this crucial structure reduces the lipophilicity of the parent structure by increasing the spatial effect. Substitute addition also breaks the symmetry of the original structure and forms a chiral center, generating a stereoselective product. These changes lead to a higher receptor affinity for ciprofol than for propofol. Furthermore, compared with the *S*-isomer of ciprofol, the *R*-enantiomer possesses better stereoselectivity for the GABA_A_ receptor and is more potent than the *S*-isomer. Ciprofol has superior advantages over propofol in terms of target selectivity, as shown in a radioligand-binding assay [16], indicating a higher intensity of action (Table 1).

Like propofol, ciprofol is a positive allosteric modulator and direct agonist of the GABA_A_ receptor. Competitive binding assays and whole-cell patch-clamp experiments demonstrated that ciprofol could trigger chloride influx by competitive binding to butylbicyclophosphorothionate and t-butylbicycloorthobenzoate targets in the chloride channels of GABA_A_ receptors [19]. The influx of chloride can cause hyperpolarization of nerve cell membranes by increasing the intracellular chloride concentration and further activating GABAergic neurons to achieve central nerve inhibition, producing sedative and anesthetic effects.

## 3. Pharmacodynamics

### 3.1. Preclinical Studies

Animal experiments have confirmed the sedative and anesthetic effects of ciprofol. Loss of righting reflex (LORR), a widely used behavioral surrogate to assess the hypnotic effects of drugs [20, 21], was observed in all animals at doses of 2.0 mg/kg and above, with durations proportional to dosage. The doses of ciprofol for generating hypnotic activity in rats at 10 min (HD_10_) and 50 min (HD_50_) after LORR were approximately one-fifth and one-sixth than that of propofol doses, respectively. The median lethal dose (LD_50_) of ciprofol was one-fourth than that of propofol, and its therapeutic index was slightly higher than that of propofol (Table 2) [19]. These results confirmed that ciprofol has obvious advantages in terms of the selectivity and affinity of the targets and may achieve the same clinical effects as propofol at lower doses.

### 3.2. Clinical Trials

As shown in Table 3, several clinical trials have confirmed the sedative and anesthetic effects of ciprofol.

#### 3.2.1. Phase I Clinical Trials

The phase I clinical trials established a safe and effective single loading dose range for intravenous injection of ciprofol emulsion. In addition, the initial maintenance dose and subsequent maintenance dose ranges of intravenous ciprofol infusion for prolonged sedation and anesthesia were also investigated, and the recommended adjustment dose range was determined.

A phase I clinical trial (*n* = 24) was conducted on healthy Chinese subjects to assess the anesthetic dose of ciprofol. The study indicated that the modified Observer's Assessment of Alertness/Sedation (MOAA/S) score decreased rapidly after administration of 0.4, 0.6, and 0.9 mg/kg of ciprofol. The corresponding occurrences of loss of verbal response (LOR_verbal_) were 83.3%, 100%, and 100%, respectively [22]. The duration of recovery of the verbal response (ROR_verbal_), LOR_verbal_, MOAA/S ≤ 1 (deep sedation), and return of responsiveness (*t*_alert_) were all prolonged with increasing dosages. In addition, the durations of the bispectral index (BIS) < 60 (unresponsive to verbal stimuli) in the three dose groups were 6, 8, and 12 min, respectively, with BIS_peak_ decreasing in a dose-dependent manner. The median times to loss of eyelash reflex were 1.7, 1.5, and 1.0 min in the three dose groups, respectively. The median orientation recovery times were 9.0, 11.0, and 19.5 min, exhibiting a significant dose dependence. The modified quality of recovery- (QoR-) 9 scores of the three groups of subjects were more than 17 points (maximum 18 points) with no significant changes before and after ciprofol administration, indicating good recovery after anesthesia. Ciprofol doses ranging from 0.4 to 0.9 mg/kg were well tolerated by patients. The result provided a recommended ciprofol dose greater than 0.4 mg/kg for phase II clinical studies.

A following phase I clinical trial (*n* = 64) was conducted to evaluate the effects of the continuous infusion of ciprofol on its pharmacodynamic and PK properties and safety profiles [23]. The effects of anesthesia were evaluated using the Richmond Agitation Sedation Scale (RASS) and BIS. The results showed that there were no statistical differences in the average time to the onset of sedation (12.58 min vs. 12.60 min) and recovery after the end of anesthesia (5.73 min vs. 6.75 min) when the subjects received the initial dose of ciprofol or propofol. Similar conclusions were obtained in the bolus dose and maintenance dose groups, except for the average duration of sedation (-3 RASS ≤ −1), in which the time of propofol was slightly longer than that of ciprofol (469.11 ± 22.2 min vs. 389.06 ± 27.7 min, *P* = 0.049). No significant differences were found in other pharmacodynamic parameters. The mean RASS-time and BIS-time curves for ciprofol were similar to those for propofol. These data suggest that ciprofol is noninferior to propofol in terms of safety, tolerability, and efficacy in maintaining sedation by continuous intravenous infusion. A dose adjustment proposal was proposed for the next phase of the study, based on the tolerance performance of the subjects. The loading dose of ciprofol was 0.1-0.2 mg/kg for 1-5 min of infusion with a dose adjustment range of 0.05-0.1 mg/kg/h. The initial maintenance dose was 0.3 mg/kg/h, with the subsequent maintenance dose 0.06-0.8 mg/kg/h and an adjusted dose range of 0.05-0.1 mg/kg/h [23].

#### 3.2.2. Phase II and III Clinical Trials

In phase II and III clinical trials, the efficacy and safety characteristics of ciprofol were compared with those of propofol, and the appropriate dose was further confirmed in different clinical scenarios.

*(1) Gastroscopy and Colonoscopy*. Multicenter phase IIa and IIb clinical trials investigated the efficacy and safety of ciprofol for sedation and anesthesia in patients undergoing colonoscopy [24]. There were 64 subjects enrolled in the phase IIa trial, and the success rates of colonoscopy insertion were 100% in the 0.2-0.5 mg/kg ciprofol and 2.0 mg/kg propofol groups, with generally well tolerated. The duration of colonoscopy insertion in the ciprofol 0.5 mg/kg group was similar to that in the propofol group (1.2 ± 0.4 min vs. 1.2 ± 0.6 min, *P* > 0.05). The recommended doses of ciprofol for subsequent phase IIb trials were 0.4 mg/kg and 0.5 mg/kg. In the phase IIb trial (*n* = 94), the success rates were 100% in the ciprofol 0.4 mg/kg and 0.5 mg/kg groups and the propofol 2.0 mg/kg group. The mean colonoscopy insertion times were 1.9, 1.5, and 1.5 min (*P* > 0.05), and the mean recovery times of colonoscopy withdrawal were 6.1, 5.1, and 4.3 min, respectively. Additionally, the satisfaction rate of the anesthetists in the 0.5 mg/kg ciprofol group (9.5 ± 0.8) was significantly higher than that in the 0.4 mg/kg ciprofol group (9.2 ± 1.0) and 2.0 mg/kg propofol group (9.2 ± 0.9) (*P* < 0.05). These results initially indicated the efficacy and safety of ciprofol for sedation or general anesthesia during colonoscopy and defined the optimal dose.

A phase III clinical trial was subsequently conducted in China to compare the effects of ciprofol and propofol on inducing deep sedation during gastroscopy (*n* = 30) and colonoscopy (*n* = 259) procedures [25]. In this trial, the success rate of colonoscopy was 100% in the ciprofol group and 99.2% in the propofol group (mean difference, 0.8%; 95% CI: 2.2% to 4.2%); the gastroscopy success rate was 100% in both groups. There were no significant differences between the two groups in terms of induction time (MOAA/S ≤ 1 after administration of the initial dose), insertion time, and insertion success rates. It is worth noting that the mean time for a patient to become fully alert in the ciprofol group was longer than the propofol group in the overall analysis, and the time to discharge of the ciprofol group was significantly longer than that of the propofol group (7.4 ± 3.1 min vs. 6.0 ± 2.1 min, *P* < 0.001), whereas patient satisfaction scores of the ciprofol group were significantly superior to that of the propofol group (9.9 ± 0.4 vs. 9.7 ± 0.7, *P* = 0.001) [25]. A larger number of subjects demonstrated ciprofol 0.4 mg/kg were noninferior to 1.5 mg/kg propofol in the success rate of gastroscopy or colonoscopy.

*(2) Fiber-Optic Bronchoscopy*. A phase III trial enrolled patients who underwent fiber-optic bronchoscopy (*n* = 267) to evaluate the efficacy, safety, and PK of ciprofol [26]. Both the ciprofol 0.4 mg/kg and propofol 2.0 mg/kg groups completed the procedure with a success rate of 100%. All subjects reached deep sedation (MOAA/S ≤ 1) or general anesthesia (95% confidence interval (CI) for the difference between the two groups was −2.8 to 2.8%). More than half of the patients in both groups underwent fiber-optic bronchoscopy without top-up dose. The median time to administration of the top-up dose was almost identical among patients who required a booster dose. The median time to successful insertion of the fiber-optic bronchoscope and induction of anesthesia/sedation was similar between the two groups (*P* > 0.05). Furthermore, the anesthesia/sedation satisfaction scores of the patients and anesthesiologists in the ciprofol group were comparable to those in the propofol group. However, the median time to full alertness (8.50 vs. 6.00 min, *P* = 0.012) and the median time to discharge (13.00 vs. 9.87 min, *P* = 0.002) were slightly longer in the ciprofol group. This multicenter, double-blind, randomized, noninferiority, parallel-group trial confirmed that ciprofol manifested anesthetic/sedative effects comparable to propofol in patients undergoing bronchoscopy, with a lower incidence of pain on injection.

*(3) General Anesthesia in Elective Surgery*. A phase II clinical trial assessed the efficacy and safety of ciprofol for the induction and maintenance of general anesthesia in elective surgical patients (*n* = 46) [27]. 40 of them were randomly assigned to the ciprofol or propofol group at a 3 : 1 ratio. The remaining six subjects received the “propofol+ciprofol” regimen. The results of the trial showed that the success rates of anesthesia induction and maintenance were 100% in all three groups, without any top-up doses or rescue therapy. This convincing finding supported that ciprofol is likely to have high efficacy in patients undergoing restricted elective surgery, even after induction with a different anesthetic drug. In addition, the onset time of the ciprofol 0.4 mg/kg group was comparable to propofol 2.0 mg/kg group, and both were successfully induced within 1 min during the induction phase. In the maintenance phase, the ciprofol group had a comparable proportion of patients whose BIS values were always maintained between 40 and 60 (13.3% vs. 10.0%, *P* = 1.000) and the proportion of durations with BIS values between 40 and 60 during the entire anesthesia maintenance period (69.9 ± 24.7% vs. 70.5 ± 18.2%, *P* = 0.948) compared to the propofol group. The time from the discontinuation of anesthetic drug maintenance to full alertness and other recovery-related durations was the same for ciprofol and propofol (all *P* > 0.05). The satisfaction score of anesthesiologists for patients receiving ciprofol was comparable to that of propofol during the maintenance period but tended to be more satisfactory during the induction period [27].

A subsequent phase III trial compared ciprofol with propofol for the successful induction of general anesthesia in patients (*n* = 176) undergoing elective surgery [28]. The success rate of anesthesia induction in both groups was 100.0% indicating that ciprofol was not inferior to propofol in the induction of general anesthesia. The mean times for successful induction of general anesthesia and loss of eyelash reflexes were 0.91 and 0.80 min for ciprofol and 0.80 and 0.71 min for propofol, respectively. Although the time was slightly prolonged in the ciprofol group, the average time to anesthesia induction in the two groups was still within 1 min. The exposure doses of ciprofol and propofol for each patient were 26.0 mg and 121.8 mg, respectively. In addition, ciprofol showed a pattern of BIS changes similar to that of propofol and was stable during the maintenance of anesthesia. The mean satisfaction scores of anesthesiologists with ciprofol and propofol were 10.9 and 10.8, respectively, with no statistically significant differences. Combining the data of the two stages of clinical trials, ciprofol is a good candidate for patients scheduled for elective surgery.

*(4) Sedation during Intensive Care Units (ICU)*. Based on previous studies, a multicenter, open-label, randomized, propofol-positive-controlled phase II trial (*n* = 39) was conducted in Chinese patients admitted to the ICU [29]. This study was designed to investigate the safety, efficacy, and PK characteristics of ciprofol for sedation in patients undergoing mechanical ventilation. Ciprofol was injected at a loading dose of 0.1-0.2 mg/kg for 0.5-5.0 min and followed by a maintenance infusion at a rate of 0.3 mg/kg/h for 6-24 h. Doses were adjusted according to RASS scores (−2 ≤ RASS ≤ +1) to maintain the target depth of sedation. The propofol loading and maintenance doses were 0.5-1 mg/kg and 1.5 mg/kg/h. The results showed that the median sedation times for ciprofol and propofol were 60.0 min. Drug-related and sedation-related treatment-emergent adverse events (TEAEs) included hypotension (7.7% vs. 23.1%, *P* = 0.310) and sinus bradycardia (3.8% vs. 7.7%, *P* = 1.000) in the ciprofol and propofol groups, respectively. The study showed that ciprofol was comparable to propofol, with good tolerance and efficacy in ICU patients on mechanical ventilation. A subsequent phase III trial (*n* = 135) is aimed at further demonstrating that ciprofol has the same efficacy as propofol and elicits fewer complications in ICU patients [30]. Based on information from *Clinicaltrials.gov*, this study has already been completed. Although no relevant study results have been posted for this trial, the conclusions of the experiment should be satisfactory, given that the drug was later approved for sedation in the ICU.

#### 3.2.3. Phase IV Clinical Trials

The value of ciprofol was further verified in clinical practice according to the recommended dose in the drug instructions approved by NMPA.

Chen et al. further compared the differences in intraoperative adverse reactions, operation, resuscitation, and satisfaction of patients between ciprofol and propofol in 96 clinical patients undergoing painless gastroenteroscopy, evaluated the clinical value of the two drugs, and obtained similar conclusions to those of previous studies [31]. The incidence of side effects was lower in the ciprofol group than in the propofol group (53.19% vs. 63.26%, *P* < 0.05). However, the mean arterial pressure (MAP) of patients in the ciprofol group was significantly lower than that of the propofol group after 1 min of administration (92.11 vs. 98.22, *P* = 0.011), and the diastolic blood pressure (DBP) of the ciprofol group was significantly lower marginal significance lower than that of the propofol group (71.75 vs. 75.22, *P* = 0.08) after 3 min of administration.

A prospective, double-blind, single-center trial enrolled 120 female patients between the ages of 18 and 60, to induce general anesthesia and complete gynecologic surgery [32]. In this study, there was no significant difference between the ciprofol and propofol groups in terms of induction success rate, the duration of successful induction, the time to the disappearance of the eyelash reflex, and tracheal intubation (all *P* values > 0.05), indicating that the efficacy of the two drugs was comparable. The overall incidence of adverse reactions was lower in the ciprofol group than in the propofol group (20% vs. 48.33%, *P* = 0.0019).

## 4. Pharmacokinetics

After intravenous injection in rats, ciprofol was found to be widely distributed in the tissues. The results showed that ciprofol could be rapidly eliminated from most tissues, thus alleviating the postanesthesia effect. Ciprofol concentrations in the adrenal gland, fat, skin, ovary, and kidneys were five times higher than plasma concentrations. Exposure in brain tissues was 3.2 times higher than that in plasma, indicating that ciprofol could easily penetrate the blood-brain barrier [19]. The time to peak concentration (*T*_max_) in most tissues was the same as that in plasma (4 min). The residual concentration of ciprofol was less than 10% of the peak concentration (*C*_max_) after 240 min of administration, except in the fat, skin, bladder, and uterus. Furthermore, ciprofol was widely bound to plasma proteins [19, 33]. The binding rate of human plasma protein was up to 95% within a concentration range of 80-1200 ng/mL (Table 4).

A PK study demonstrated that after a single dose of intravenous injection of C^14^-labeled ciprofol (0.4 mg/kg) in healthy subjects, *T*_max_ was 2-3 min and decreased to near baseline within approximately 10 min. Ciprofol is metabolized in the liver through cytochrome P450 (CYP) 2B6 and uridine diphosphate-glucuronyltransferase (UGT) enzymes. Based on these metabolites, the main in vivo metabolic pathways of ciprofol were proposed to be oxidation → glucuronic acid binding → sulfuric acid binding. The primary metabolite was a ciprofol-glucuronic acid conjugate, and the secondary metabolite was a ciprofol-monooxidized glucuronic acid conjugate. Ciprofol and its metabolites are excreted primarily through the urine (84.6%) and feces (2.65%) [34].

One study evaluated ciprofol PK parameters under different administration models in healthy subjects (single-dose administration, sequential maintenance administration after the initial dose, and maintenance dose after the loading dose). The results are presented in Table 5. After a single 0.4 mg/kg dose of ciprofol, the following PK parameters were obtained: *C*_max_ 1,330.0 ng/mL, *T*_max_ 2.0 min, the elimination half-life (*T*_1/2_) 2.09 h, the clearance (CL) 1.47 L/h/kg, the area under the curve (AUC_0−∞_) 271.67 ng·h/mL, and the apparent volume of distribution (Vd) 4.3 L/kg [22]. These PK parameters showed a rapid onset of action and short peak time after a single dose of ciprofol. Furthermore, the drug had a high CL and small Vd, consistent with the clinical expectations of short-term intravenous anesthesia. Consequently, when the maintenance dose was continued after the initial or loading dose of ciprofol, both *T*_max_ and *T*_1/2_ were significantly prolonged, with a greater Vd. Ciprofol has the potential for clinical application as a continuous intravenous infusion to maintain sedation for 12 h [23].

The pharmacokinetic properties of ciprofol are virtually similar to those of propofol, as demonstrated in a phase II clinical trial of the induction and maintenance of general anesthesia in elective surgical patients. During the anesthesia maintenance phase, the plasma concentration of ciprofol in the propofol+ciprofol group was the same as that in the ciprofol group, and the same pattern of propofol concentration was evident in the propofol group during the anesthesia induction period [27]. Propofol was 4–5 times higher than ciprofol in terms of drug exposure. The *T*_1/2_ (13.0 ± 10.9 h vs. 19.6 ± 2.2 h) and Vd (18.0 ± 18.8 L vs. 32.0 ± 9.3 L) of ciprofol were slightly lower than propofol, and the *T*_max_ (0.17 h vs. 0.11 h) and CL (1.0 ± 0.4 L/h/kg vs. 1.1 ± 0.3 L/h/kg) values were very similar [27].

## 5. Safety

The reverse mutation (*Ames*) test with *Salmonella typhimurium*, chromosome aberration test with Chinese hamster lung fibroblasts, and mouse bone marrow micronucleus test showed negative genotoxicity findings. Furthermore, no apparent reproductive toxicity of ciprofol was observed in rat fertility and early embryonic development toxicity tests, embryo-fetal developmental toxicity tests, and perinatal developmental toxicity tests. The effects on the respiratory and cardiovascular systems were evaluated by telemetry after intravenous bolus injection of control solvent or ciprofol (1, 2, or 4 mg/kg) in beagle dogs [19]. The results showed that transient tachycardia occurred two minutes after injection in all groups. Heart rates increased by 88%, 106%, 134%, and 169%, respectively, which could be due to nervousness of the animals after injection. There was no significant change in respiratory rate after ciprofol administration. However, the corrected QT interval (QTc) significantly lengthened within one hour of administration in a dose-dependent manner. Simultaneously, body temperature significantly decreased, with the 4 mg/kg group showing the most significant decrease among the three groups. Furthermore, after administering 2 mg/kg or 4 mg/kg ciprofol, blood pressure was significantly reduced in one hour, with systolic blood pressure and diastolic blood pressure decreasing by 18.8% and 23.3%, respectively. The mean arterial pressure decreased by 21.3%. No after-effects were observed in the recovered animals.

Ciprofol is generally well tolerated in clinical trials. Common ADRs include hypotension, bradycardia, apnea, respiratory depression, hypoxia, and pain during injection. Except for five cases of serious ADR in the phase IIa trial of sedation or anesthesia in patients undergoing colonoscopy and the phase III trial of induction of deep sedation during gastroscopy and colonoscopy procedures [24, 25], other ADRs were mild or moderate, in which patients could recover after brief treatment or without interventions. With the exception of one subject who withdrew due to epilepsy in a phase II trial of sedation in ICU patients with mechanical ventilation, there was no withdrawal from the clinical trials due to ADRs. No fatal ADRs occurred, and none of the common ADRs of ciprofol were dose-dependent. Compared to propofol (2 mg/kg), ciprofol (0.4 mg/kg) had a lower incidence of TEAEs (Table 6). Combining all the reported clinical trial data, ciprofol was comparable to propofol in terms of the incidence of hypotension, bradycardia, and QT interval prolongation. In phase III trials of colonoscopy and gastroscopy sedation, the incidence of respiratory depression was significantly lower in the ciprofol group than in the propofol group (2.8% vs. 5.5%) [25].

As a common TEAE of propofol, the incidence of pain on injection decreased dramatically with ciprofol (Table 6). The free drug in the aqueous propofol solution directly contacts the nerve endings in the inner wall of the blood vessels or produces pain-causing substances [35], affecting the stability of anesthesia induction. Previous studies have suggested that injection pain decreases as propofol concentration in the aqueous phase of the emulsion decreases [36]. The plasma drug concentration of ciprofol is lower than that of propofol because of its high potency. Interestingly, the free drug concentration of ciprofol is lower than that of propofol (1.06 vs. 8.28 *μ*g/mL), which is attributed to its stronger hydrophobicity at the same concentrations and conditions [24]. These properties reduce the production of substances that cause vascular pain by ciprofol, thus reducing the risk of injection pain.

Propofol dosage and lipid carrier were closely related to the increased risk of hypertriglyceridemia complications in ICU patients [37]. High-dose propofol infusion can lead to increased fat transport and hypertriglyceridemia. The propofol solution (1%) contains 10% soybean oil, 2.25% glycerin, and 1.2% refined egg phospholipids [10, 38]. In contrast, the concentrations of these three substances in the ciprofol solution (1%) were 5%, 2.25%, and 1.2%, respectively. The lower fat content of ciprofol may decrease the incidence of triglyceridemia. However, further studies are warranted to evaluate the effects of ciprofol on hypertriglyceridemia.

Although the safety of ciprofol is significantly superior to that of propofol, the adverse reaction of muscle fasciculation still raises our concern. Abnormal limb movements, including muscle fasciculation, were first observed in a dose-dependent manner in a phase I clinical trial. The incidence in the 0.4, 0.6, and 0.9 mg/kg groups was 33.3%, 33.3%, and 83.3%, respectively [22]. The subsequent colonoscopy sedation/anesthesia safety trial suggested a higher incidence of adverse effects of ciprofol in eliciting muscle fasciculation than that of propofol [24]. In the phase IIa trial, the incidence of muscle fasciculation in the 0.4 mg/kg ciprofol group was higher than that in the 2.0 mg/kg propofol group (4.5% vs. 0%). In the subsequent phase IIb trial, muscle fasciculation was observed only in the 0.2 mg/kg ciprofol group and was not dose-dependent. Consequently, researchers have speculated that muscle fasciculation may be related to low doses of anesthetic drugs, as anesthesia-related seizures are common during induction or when the concentration of an anesthetic drug is relatively low [39]. In a phase II trial of sedation in ICU patients with mechanical ventilation and a phase III trial of general anesthesia induction in patients for elective surgery, one case of epilepsy and one case of myoclonus have been reported [28, 29]. Combining the results of these four clinical trials, muscle fasciculation occurred at various doses (0.2, 0.3, 0.4, and 0.9 mg/kg) of ciprofol, mainly during the induction of sedation/anesthesia. In contrast, no adverse reactions were observed in the control group. As there is no reliable evidence on the mechanism of ciprofol-induced muscle fasciculation, further research should be performed in the future.

In summary, ciprofol has noninferior effects on heart rate and blood pressure compared to propofol, with less respiratory depression, extremely low incidence of pain on injection, and less lipid input. However, fascicular fibrillation still requires further investigation.

## 6. Special Population

**Figure 2:**
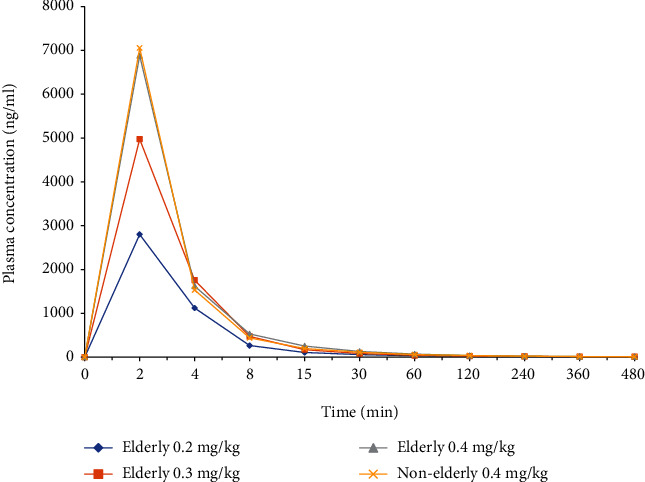
Plasma concentration-time curves of ciprofol in the elderly and nonelderly patients.

The pharmacodynamic and PK properties and safety of ciprofol were investigated in healthy elderly and nonelderly Chinese adults [40]. Elderly subjects (*n* = 24, 65-73 years old) were randomly assigned to three experimental groups to receive a single intravenous dose of ciprofol at 0.2, 0.3, and 0.4 mg/kg, respectively. In the control group, nonelderly subjects (*n* = 8, 21-44 years old) were given a dose of 0.4 mg/kg. The results demonstrated that after intravenous infusion of ciprofol, the plasma exposure level in the elderly group increased with increasing doses. The distribution and elimination of drugs were not significantly different between the groups. The PK profile of ciprofol (0.4 mg/kg was comparable between the two groups, suggesting that age had no significant effect on plasma ciprofol exposure (Figure 2). The average plasma protein-binding rates of the three experimental and control cohorts were 99.2%, 99.2%, 99.2%, and 99.1%, respectively, and the average free fractions of ciprofol in the plasma were 0.8%, 0.8%, 0.8%, and 0.9%, respectively. However, these differences were not statistically significant [40]. Compared with the other three groups, the elderly group (0.2 mg/kg) had a slightly longer time of unconsciousness and a shorter recovery time. The recovery times of the 0.3 mg/kg and 0.4 mg/kg elderly groups were similar to those of the nonelderly group. The incidence of TEAEs was slightly higher in the elderly group than that in the nonelderly group. TEAEs were slightly higher in the 0.4 mg/kg group than in the low-dose group in elderly subjects (Table 7). Considering that the 0.3 mg/kg group was comparable to the 0.4 mg/kg group for sedation and anesthesia in the elderly groups with lower adverse reactions, 0.3 mg/kg is recommended for elderly patients.

According to *Clinicaltrials.gov* and the Chinese product instruction by NMPA, mild and moderate hepatic insufficiency had little effect on the efficacy of ciprofol (MOAA/S, BIS). The safety profile after a single intravenous infusion of ciprofol in subjects with mild and moderate hepatic impairment was good, no SAEs were reported, and no subjects were withdrawn from the trial due to AEs. Clinical intravenous infusion of ciprofol for patients with mild and moderate hepatic insufficiency does not require dose adjustment; however, safety monitoring needs to be strengthened. Likewise, the degree of renal impairment had little effect on the pharmacodynamic indicators (MOAA/S and BIS) of ciprofol because of the high protein-binding rate. The safety of ciprofol in patients with mild to moderate renal insufficiency has also been confirmed; no SAEs were reported, no subjects were withdrawn from the trial due to AEs, and no dose adjustment was necessary in clinical settings. Owing to the lack of clinical research data in patients with severe hepatic or renal deficiencies or those requiring dialysis, ciprofol is not recommended for such patients. Qin et al. [41] compared the efficacy and safety of ciprofol and propofol in 120 patients undergoing general anesthesia for kidney transplantation. The results showed that the sedation success rate was 100% in both groups. Compared with the propofol group, the time to disappearance of the eyelash reflex and the time for BIS to drop to 60 in the ciprofol group were shorter, the recovery time was longer, and the intraoperative sedative drug dosage was lower (all *P* values < 0.001). The incidence of pain on injection and intraoperative hypotension in the ciprofol group was lower than that in the propofol group (*P* < 0.01), and there was no significant difference in the occurrence of other adverse reactions. The early postoperative renal transplantation function of both groups seemed to show significant improvement, with no significant difference in the terms of kidney function recovery index, indicating that ciprofol can be safely used in kidney transplant patients.

There are no clinical research data on ciprofol in patients under 18 years old and pregnant and lactating women. Clinicians must evaluate the benefits and risks to pregnant women when considering the use of ciprofol; lactating women should stop breastfeeding while on this drug. Given that propofol infusion syndrome was first reported in pediatric patients [42, 43], ciprofol is not recommended for sedation and anesthesia in pediatric patients until its use and dosage have been fully validated and approved.

## 7. Drug-Drug Interaction

There were no pharmacological incompatibilities of ciprofol in combination with fentanyl, sufentanil, or midazolam, and the effects were satisfactory. A study compared the anesthetic effects of ciprofol alone or in combination with low-dose sufentanil in painless gastroscopy [44]. The results showed that, compared with the single group, the total dosage of ciprofol in the combined group was significantly reduced, the induction time was shorter (*P* < 0.05), and the overall incidence of adverse events was significantly lower (*P* < 0.001). In addition, a single-center, randomized, double-blind, placebo-controlled, 2 × 2 factorial clinical trial was conducted in China to evaluate the effects of esketamine in combination with ciprofol or propofol on respiratory and hemodynamic adverse events in patients with the aim of providing evidence for daily practice of sedation regimens for same-day bidirectional endoscopy [45]. Although the clinical trial is still in progress, relevant data have not been reported. However, as an integral part of this trial, the drug-drug interaction between esketamine and ciprofol will also be examined. Whether any pharmacological incompatibility exists between the two drugs and the safety of the combination will be fully validated in future results.

## 8. Conclusions

Ciprofol is currently approved for sedation and anesthesia during the surgical/procedure of nontracheal intubation, induction and maintenance of general anesthesia, and sedation during intensive care in China. Owing to these structural modifications, the binding ability of ciprofol to GABA_A_ receptors was 4-5 times higher than that of propofol in whole-cell patch-clamp experiments. The higher selective binding ability of ciprofol to the receptors enables it to achieve the same sedative and anesthetic effects as propofol at a lower dosage. In preclinical experiments, the therapeutic index of ciprofol in rats was slightly higher than that of propofol, indicating that ciprofol has a wider safety window. In addition, the results of multiple clinical trials on ciprofol further confirmed that this drug has advantages such as better tolerance, higher sedation satisfaction score, and lower incidence of adverse reactions, especially in effectively reducing the incidence of pain on injection. Regrettably, ciprofol has been on the market for a short period and still lacks long-term medication experience and clinical data validation of broader clinical scenarios. Additionally, the lack of evidence for drug safety in special populations severely limits the wider application of ciprofol. Anesthesiologists are more proficient and accustomed to using propofol, and it is much too ambitious to consider that propofol is completely replaced by ciprofol at present. Even when dealing with adverse reactions to propofol, anesthesiologists can deal with it more calmly. Therefore, it remains the preferred choice for such drugs in clinical intravenous anesthesia. Nonetheless, the safety advantage of ciprofol may provide a more stable anesthesia process for the clinic and effectively reduce and alleviate postoperative complications in patients, especially in the elderly. With the deepening research on ciprofol, it is reasonable to expect that this drug will become a novel alternative for clinical intravenous anesthesia and will bring more benefits to patients.

## Figures and Tables

**Table 1:** Results of GABA_A_ receptor binding analysis of propofol and ciprofol.

Compound	Conformation	Binding assay (% inhibition)	
		10 *μ*M	1 *μ*M
Propofol	—	~10	/
Ciprofol	Mix	3	-12
	*R*	85	34
	*S*	-9	3

Notes: data from Qin et al. [16].

**Table 2:** Dosage comparison of ciprofol and propofol on LORR in rats.

Test group	Dosage (mg/kg)			TI
	HD_50_	LD_50_	HD_10min_	
Ciprofol	0.88	8.00	1.84	9.1
Propofol	5.05	31.31	11.50	6.2

Notes: data from Liao et al. [19]. Abbreviations: HD_50_: median hypnotic dose; LD_50_: median lethal dose; HD_10min_: dose for maintaining 10 min hypnosis; TI: therapeutic index; TI = LD_50_/ED_50_.

**Table 3:** Comparison of sedative/anesthetic pharmacodynamics between ciprofol and propofol.

Clinical trials	Phase	Number enrolled	Groups	Dose (mg/kg)			*t*_sedation_ (min)			*t*_alert_ (min)			Sedation satisfaction score		
				Mean ± SD	Median (min, max)	*P*	Mean ± SD	Median (min, max)	*P*	Mean ± SD	Median (min, max)	*P*	Mean ± SD	Median (min, max)	*P*
Phase I clinical study in healthy subjects [23]	I	8	Ciprofol (iv, 1 mg/kg/h for 0.5 h + 0.4 mg/kg/h for 3.5 h)	2.05 ± 0.45	—	—	12.58 ± 8.79	—	0.917	5.73 ± 2.34	—	0.463	6.8 ± 1.20	—	0.041
		8	Propofol (iv, 5 mg/kg/h for 0.5 h + 2 mg/kg/h for 3.5 h)	10.88 ± 1.62			12.60 ± 6.90			6.75 ± 1.61			5.0 ± 2.10		
Anesthesia/sedation in patients undergoing colonoscopy [24]	IIa	10	Ciprofol (iv, 0.4 mg/kg)	—	—	—	1.6 ± 0.5	—	>0.05	6.0 ± 3.3^a^	—	<0.05	10.0 ± 0.0	—	
		10	Ciprofol (iv, 0.5 mg/kg)				1.2 ± 0.4			10.3 ± 5.6^a^			9.9 ± 0.3		
		10	Propofol (iv, 2 mg/kg)				1.2 ± 0.6			5.0 ± 3.3^a^			9.6 ± 0.7		
	IIb	31	Ciprofol (iv, 0.4 mg/kg)	—	—	—	1.9 ± 0.6	—	>0.05	6.1 ± 4.5^a^	—	—	9.2 ± 1.0	—	<0.05
		32	Ciprofol (iv, 0.5 mg/kg)				1.5 ± 0.3			5.1 ± 2.9^a^			9.5 ± 0.8		
		31	Propofol (iv, 2 mg/kg)				1.5 ± 0.4			4.3 ± 3.3^a^			9.2 ± 0.9		
Induction of deep sedation during gastroscopy and colonoscopy procedures [25]	III	144	Ciprofol (iv, 0.4 mg/kg)	—	—	—	1.1 ± 0.5	—	0.405	3.3 ± 3.1	—	<0.001	9.9 ± 0.4	—	0.001
		145	Propofol (iv, 1.5 mg/kg)				1.1 ± 0.4			2.0 ± 2.1			9.7 ± 0.7		
Anesthesia/sedation in patients undergoing fiber-optic bronchoscopy [26]	III	134	Ciprofol (iv, 0.4 mg/kg)	—	26.75 (14.75, 79.50)	<0.001	—	1.00 (0.50, 3.50)	0.059	—	8.50 (0.10, 27.90)	0.012	—	10 (6, 10)	0.341
		133	Propofol (iv, 2 mg/kg)		142.00 (26.75, 549.00)			1.00 (0.45, 8.00)			6.00 (0.00, 29.00)			10 (4, 10)	
Induction/maintenance of general anesthesia in elective surgery [27, 28]	II	30	Ciprofol (iv, 0.4 mg/kg + 1 mg/kg/h for 6 patients and 0.8 mg/kg/h for 24 patients)	—	—	—	45.3 ± 14.8	—	0.840^c^	11.4 ± 4.9	—	0.575^c^	11.8 ± 0.8^d^ 11.3 ± 1.1^e^	—	0.024^d^ 0.814^e^
		10	Propofol (iv, 2 mg/kg + 5 mg/kg/h for 2 patients and 6 mg/kg/h for 8 patients)				42.3 ± 15.3			11.8 ± 3.4			10.6 ± 2.4^d^ 11.4 ± 1.3^e^		
		6	Propofol (iv, 2 mg/kg)+ciprofol (iv, 1 mg/kg/h)				56.7 ± 13.7			13.5 ± 4.6			11.2 ± 2.0^d^ 10.3 ± 2.0^e^		
	III	88	Ciprofol (0.4 mg/kg, bolus iv over 30 s)	26.0 ± 5.8^b^	—	—	0.91 ± 0.03	—	<0.05	—	—	—	10.9 ± 1.4	—	0.496
		88	Propofol (2 mg/kg, bolus iv over 30 s)	121.8 ± 24.6^b^			0.80 ± 0.03						10.8 ± 1.4		
Sedation in ICU patients with mechanical ventilation [29, 30]	II	26	Ciprofol (iv, 0.1-0.2 mg/kg/h for 0.5 − 5 min + 0.3 mg/kg/h)	—	0.3 (0.1, 0.5)	<0.001	—	60.0 (52.6, 60.0)	—	—	4.0 (0.0, 29.4)	0.418	—	1.0 (1.0, 2.0)^f^	0.521
		13	Propofol (iv, 0.5-1 mg/kg/h for 0.5 − 5 min + 1.5 mg/kg/h)		1.5 (1.1, 1.6)			60.0 (55.2, 60.0)			4.5 (3.3, 24.6)			1.0 (1.0, 2.0)^f^	

Notes: ^a^time to alert from colonoscope withdrawal; ^b^total dose (mg); ^c^ciprofol group vs. propofol group; ^d^induction period; ^e^maintenance period; ^f^nursing score, overall evaluation of adaptive capacity for endotracheal intubation and mechanical ventilation; — indicates the data is not available. Abbreviations: *t*_sedation_: time to onset of anesthesia/sedation; *t*_alert_: time to full alertness or recovery from anesthesia/sedation.

**Table 4:** Plasma protein-binding rate of ciprofol in humans, beagle dogs, and rats.

Plasma proteins	Binding rate (%)		
	80 ng/mL	400 ng/mL	1200 ng/mL
Human	96.6 ± 0.4	94.6 ± 0.5	93.5 ± 0.3
Dog	87.9 ± 0.7	91.1 ± 0.2	93.4 ± 0.5
Rat	94.3 ± 0.9	94.9 ± 0.2	85.3 ± 0.1

Notes: data from Liao et al. [19].

**Table 5:** PK parameters of single-dose injection or maintenance dosing after ciprofol infusion.

PK parameters	Dosage		
	Single dose of 0.4 mg/kg [22] median (Q1, Q3)	Initial infusion 1 mg/kg/h for 0.5 h + 0.4 mg/kg/h for 3.5 h [23] mean (CV%)	Bolus-loading dose 0.4 mg/kg + 0.4 mg/kg/h for 12 h [23] mean (CV%)
*C*_max_ (ng/mL)	1330.0 (985.0, 1710.0)	550.7 (13.4)	1282.4 (83.9)
AUC_0–t_ (ng·h/mL)	254.33 (226.67, 423.33)	2496.4 (46.3)	4417.9 (12.7)
AUC_0−∞_ (ng·h/mL)	271.67 (245.00, 505.00)	2211.6 (16.5)	4647.2 (13.2)
*T*_max_ (min)	2.00 (2.00, 3.00)	55.20 (30.00, 720.00)^#^	3.60 (1.80, 17.40)^#^
*T*_1/2_ (h)	2.09 (1.86, 2.47)	12.78 (41.7)	9.91 (17.3)
CL (L/h/kg)	1.47 (0.79, 1.63)	1.00 (19.0)	1.09 (22.9)
Vd (L/kg)	4.3 (2.1, 5.0)	18.94 (49.7)	15.45 (21.7)
*λ*_*z*_ (L/h)	0.34 (0.28, 0.37)	0.061 (32.8)	0.072 (15.3)
MRT (h)	2.36 (1.89, 2.37)	3.68 (62.5)	3.31 (16.9)

Notes: ^#^these data of *T*_max_ are presented as median (range). Abbreviations: PK: pharmacokinetics; *C*_max_: maximum plasma concentration; AUC: area under the curve; CV: coefficient of variation; *T*_max_: time of maximum plasma concentration; *T*_1/2_: terminal elimination half-life; CL: clearance after adjusting for weight; Vd: apparent volume of distribution; *λ*_*z*_: elimination rate constant; MRT: mean residence time.

**Table 6:** Incidence of adverse events of ciprofol and propofol.

Clinical trials	Groups	Any TEAEs (*n*, %)	*P*	Drug-related TEAEs (*n*, %)	*P*	Pain on injection (*n*, %)	*P*
Phase I clinical study in healthy subjects [23]	Ciprofol (*N* = 8)	6 (75.0)	—	5 (62.5)	—	1 (12.5)	—
	Propofol (*N* = 8)	8 (100)		8 (100)		7 (87.5)	
Sedation/anesthesia in patients undergoing colonoscopy [24]	Ciprofol (*N* = 31)	26 (83.9)	0.300	15 (48.4)	0.133	4 (12.9)	—
	Propofol (*N* = 32)	26 (83.9)		19 (61.3)		14 (45.2)	
Induction of deep sedation during gastroscopy and colonoscopy procedures [25]	Ciprofol (*N* = 144)	109 (75.7)	0.201	41 (28.5)	0.403	7 (4.9)	<0.001
	Propofol (*N* = 145)	100 (69.0)		35 (24.1)		76 (52.4)	
Anesthesia/sedation in patients undergoing fiber-optic bronchoscopy [26]	Ciprofol (*N* = 134)	71 (52.6)	<0.001	50 (37.0)	<0.001	6 (4.4)	<0.001
	Propofol (*N* = 133)	101 (76.5)		93 (70.5)		52, (39.4)	
Induction and maintenance of general anesthesia in elective surgical patients [27]	Ciprofol (*N* = 30)	30 (100)	—	23 (76.7)	—	—	—
	Propofol (*N* = 10)	9 (90.0)		8 (80.0)			
	Propofol+ciprofol (*N* = 6)	6 (100)		4 (66.7)			
Anesthesia in patients scheduled for elective surgery [28]	Ciprofol (*N* = 88)	78 (88.6)	0.160	70 (79.4)	—	6 (6.8)	0.014
	Propofol (*N* = 88)	84 (95.5)		79 (89.7)		18 (20.5)	
Sedation in ICU patients with mechanical ventilation [29]	Ciprofol (*N* = 26)	17 (65.4)	0.276	2 (7.7)	0.153	—	—
	Propofol (*N* = 13)	11 (84.6)		4 (30.8)		—	

Abbreviations: TEAEs: treatment-emergent adverse events.

**Table 7:** Pharmacodynamics and safety in elderly and nonelderly subjects.

Group	Dosage (mg/kg)	The median loss-of-consciousness time (min)	Mean BIS_peak_	The median times to full alertness (min)	Adverse events *N* (%)
Elderly	0.2	2.54	60.1	6.02	3 (37.5)
	0.3	2.07	58.5	14.01	2 (25.0)
	0.4	1.13	58.1	11.99	4 (50.0)
Nonelderly	0.4	1.15	48.5	10.03	1 (12.5)

Notes: data from Li et al. [40]. Abbreviations: BIS_peak_: peak bispectral index value.

## Data Availability

No data is available for this study.
